# Hemophagocytic syndrome associated with *Mycobacterium bovis* in a patient with X-SCID: a case report

**DOI:** 10.1186/s12879-020-05421-9

**Published:** 2020-09-29

**Authors:** Buyun Shi, Ming Chen, Zhi Xia, Shuna Xiao, Wen Tang, Chenguang Qin, Ying Cheng, Tao Huang, Chengjiao Huang, Yong Li, Hui Xu

**Affiliations:** 1 Department of Pediatric Intensive Care Unit (PICU), Maternal and Child Health Hospital of Hubei Province (Women and Children’s Hospital of Hubei Province), NO.745 Wu LuoRoad, Hongshan District, Wuhan City, 430070 Hubei Province China; 2 Department of Dermatology, Maternal and Child Health Hospital of Hubei Province (Women and Children’s Hospital of Hubei Province), NO.745 Wu LuoRoad, Hongshan District, Wuhan City, 430070 Hubei Province China

**Keywords:** X-SCID, *IL2RG*, Hemophagocytic syndrome, *Mycobacterium bovis*

## Abstract

**Background:**

*Mycobacterium bovis* could infect patients with immunodeficiency or immunosuppressive conditions via Bacillus Calmette-Guérin (BCG) vaccination. Tuberculosis-related hemophagocytic syndrome (HPS) is reported, but not HPS caused by *Mycobacterium bovis* in children.

**Case presentation:**

A 4-month Chinese boy presented fever and cough. The initial laboratory investigation showed the lymphocyte count of 0.97 × 10^9^/L, which decreased gradually. HPS was diagnosed based on the test results that fulfilled the HLH-2004 criteria. In addition, *Mycobacterium tuberculosis* complex was detected from his peripheral blood via metagenomic next-generation sequencing (mNGS) and *M. bovis* was identified by polymerase chain reaction-reverse dot blot (PCR-RDB). Thus, the patient was treated with Isoniazid, Rifampin, and Pyrazinamide, but not improved. However, parents refused to accept further therapy, and was discharged on the day 12 of admission. To confirm the pathogenesis, genetic analysis was performed. Mutation in the interleukin-2 receptor subunit gamma gene: Exon 6: c.854G > A; p. Arg285Gln was detected in the patient and the mother, which could underlie X-linked severe combined immunodeficiency.

**Conclusions:**

A boy with X-SCID was diagnosed with *M. bovis*-associated HPS, emphasizing that X-SCID should be considered when *M. bovis* is detected in a male infant with low lymphocyte counts.

## Background

Hemophagocytic syndrome (HPS) is a rare life-threatening disease, characterized by overactive immune system. Primary HPS is caused by mutations in genes, such as *PFR1, UNC13D,* and *STX11*, while secondary HPS is triggered by variety of conditions and may occur at any age [1]. The most common causes of secondary HPS are viral infection, bacterial infection, and autoimmune disease. However, HPS induced by *Mycobacterium tuberculosis* complex infection is extremely rare, and HPS related to *M. bovis* in children is not yet reported [2, 3]. X-linked severe combined immunodeficiency (X-SCID) is also a rare, life-threatening immunodeficiency disease caused by genes mutations [4]. Thus, an extremely rare case of patient with X-SCID also presenting HPS due to *M. bovis* has been reported in this study.

## Case presentation

**Fig. 1:**
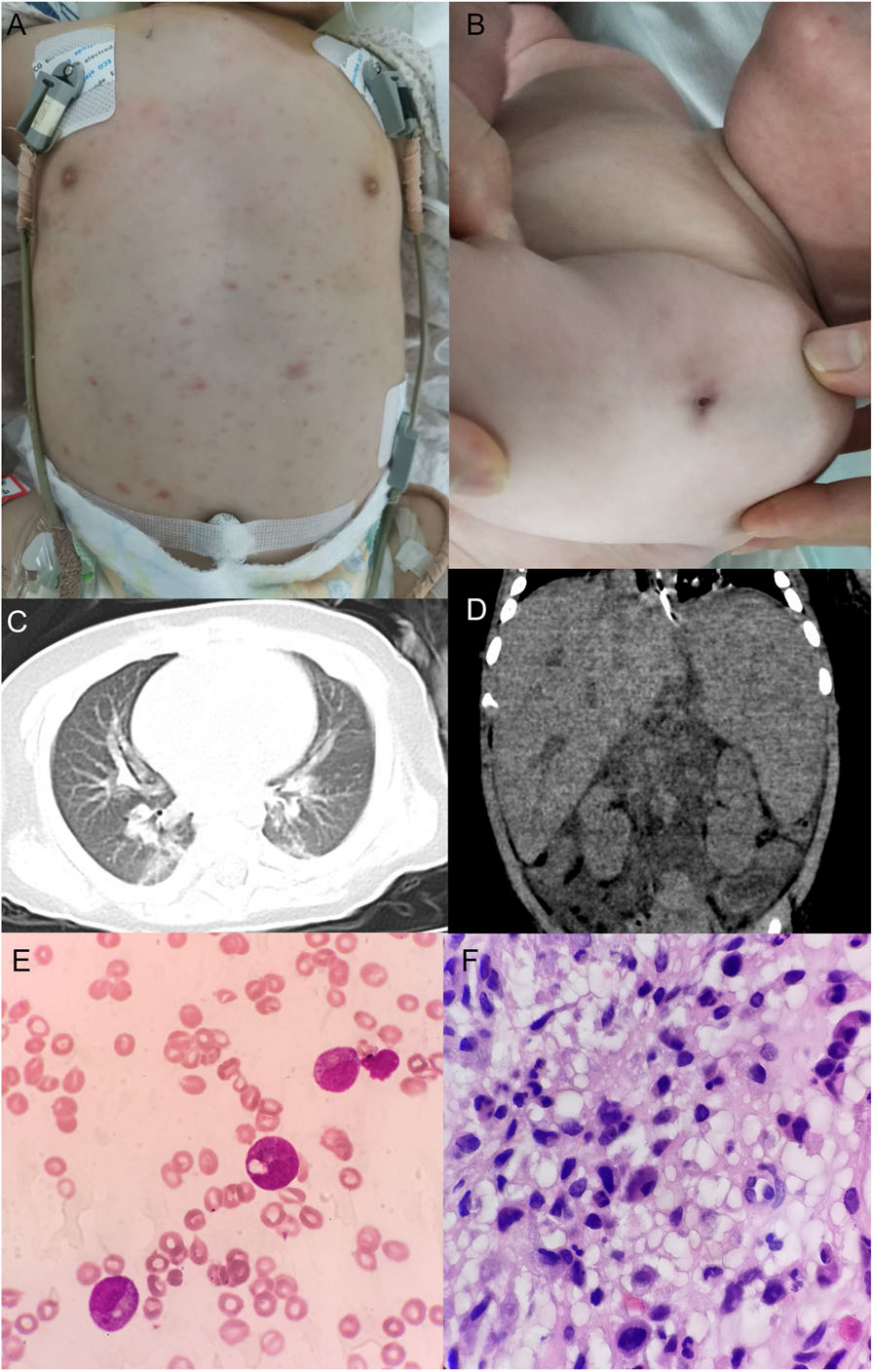
**a** Papule on the trunk. **b** Unhealed BCG scar on the left arm. **c** Chest CT scan revealed infectious lesions scattered in lungs. **d** Abdomen CT scan showed enlarged liver and spleen. **e** No hemophagocytosis was observed by bone marrow aspiration via hematoxylin and eosin (H&E) staining, high power view (× 400), lack of lymphocytes, but toxic granulation and vacuolus in neutrophils, which are signs of severe infection. **f** Numerous inflammatory cells, stained via H&E staining, high power view (× 400), were shown by bone marrow biopsy, but without evidence of malignancy

A 4-month 3-day old Chinese boy was admitted to Maternal and Child Health Hospital of Hubei Province (Women and Children’s Hospital of Hubei Province), Wuhan, in China, on May 16th, 2019. He presented intermittent fever for 5 days before admission, with the highest temperature of 39 °C accompanied by cough and papule distributed on his trunk (Fig. 1a). The symptoms worsened gradually 2 days before the admission, following which, he was diagnosed with “pneumonia” and treated with intravenous Ceftriaxone for 2 days in a local hospital. However, the symptoms did not improve significantly. Then, he was admitted to Pediatric Intensive Care Unit (PICU) at our hospital. The boy was born at term, and the BCG vaccine was administered on birth. The patient had no asthma, rash, arthralgia or relevant family history. Moreover, his 6-year-old sister was in good health. Upon admission (Day 1), skin lesions were observed on the left arm at site of BCG vaccine injection, which had not healed (Fig. 1b). Moreover, the cardiac examination was normal, his abdomen was soft, but the initial blood test showed (Table 1): white blood cells (WBC) 2.80 × 10^9^/L (normal = 4–10 × 10^9^/L), lymphocyte (L) 0.97 × 10^9^/L (normal = 1–3 × 10^9^/L), neutrophil (N) 1.59 × 10^9^/L (normal = 2–6 × 10^9^/L). Immunoglobulin profile was within the lower level, with the exception of IgG, which were slightly decrease, in particular, immunoglobulin profile and complement resulted as follows: IgG 2.1 g/L (normal = 2.6–6.9 g/L), IgM 0.28 g/L (normal = 0.26–1 g/L), IgA 0.08 g/L (normal = 0.08–0.57 g/L), C3 1.47 g/L (normal = 0.65–1.52 g/L), and C4 0.36 g/L (normal = 0.16–0.38 g/L). A chest computed tomography (CT) scan, revealed infectious lesions scattered in the both lungs (Fig. 1c). Thus, severe pneumonia was diagnosed and sepsis suspected. Then, the patient received non-invasive continuous positive airway pressure (CPAP) ventilation immediately after admission. Empric antibiotic treatment with intravenous Vancomycin (10 mg/kg every 6 h) and Meropenem (20 mg/kg every 6 h) was started after both the venous blood sample and the sputum were sent for culturing.

**Table 1:** Regular blood tests after admission

Admission day	WBC (× 10^9^/L)	N (× 10^9^/L)	L (× 10^9^/L)	HBG (g/L)	PLT (×10^9^/L)
Day 1	2.8	1.59	0.97	69	186
Day 2	1.69	1.08	0.51	52	129
Day 3	2.97	2.51	0.34	72	82
Day 4	2.23	2.01	0.13	91	91
Day 5	1.63	1.21	0.33	87	41
Day 6	1.75	1.45	0.25	75	27
Day 7	1.5	1.41	0.06	71	50
Day 8	0.46	0.41	0.04	56	27
Day 10	0.78	0.71	0.05	68	38
Day 12	0.79	0.72	0.07	56	23

The blood tests showed peripheral pancytopenia and the lymphocyte counts were low

On day 3, dyspnea was improved, but the patient still had fever, and the mental state was worsened. The level of ferritin in the serum was 2039.00 mmol/L (normal = 23.9–336.2 ng/mL). Cytomegalovirus-IgM and IgG in serum was negative. Nonetheless, pathogen was not detected in the culture of blood and sputum, and tuberculosis antibody in the serum was negative. Therefore, 3 mL of peripheral blood of the patient was sent to Guangzhou Sagene Biotech Co. Ltd. for metagenomic next-generation sequencing (mNGS). Given on persistent low lymphocyte count detected in the first 3 days (Table 1), immunodeficiency disease was suspected. Thus, after examination by the Hospital Ethics Committee and informed consent of the parents, 2 mL of peripheral blood of each of the family members, was sent to Chigene (Beijing) Translational Medical Research Center Co., Ltd. for genetic analysis for whole exome sequencing. In addition, intravenous immunoglobulin (IVIG) (1 g/kg/day, in 2 doses) was administered.

On day 6, *M. tuberculosis* complex (24,428 copies) was detected via mNGS. Subsequently, the treatment of Vancomycin and Meropenem was stopped, and Isoniazid (10 mg/kg/day, IV daily in one dose), Rifampin (5 mg/kg/day, PO every 12 h), and Pyrazinamide (10 mg/kg/day, PO every 12 h) were administered. Before starting the anti-tuberculosis therapy, 3 mL of peripheral blood was sent to Wuhan Institute for Tuberculosis Control, and *M. bovis* was identified by polymerase chain reaction-reverse dot blot (PCR-RDB).

On day 8, the patient still had high fever and splenomegaly. The abdominal CT scan showed splenomegaly, hepatomegaly, and abdominal dropsy (Fig. 1d). However, Gram-staining and acid-fast staining by abdominocentesis were negative and the culture was negative. In addition, the level of fibrinogen in blood was decreased to 0.91 g/L, and that of ferritin in the serum was increased to 3235 ng/mL. The enzyme-linked immunosorbent assay (ELISA) revealed the level of soluble CD25 cells 5182.51 pg/mL (normal = 410–2623 pg/mL). The percentage of CD3^−^CD56^+^NK cells in the lymphocyte count, assessed by flow cytometry (FCM), was 0.16% (normal range 5–26) %, granzyme B (GrB) was 80% (normal range > 78%) and perforin 86.67% (normal range > 84%). However, NK cell activity test was not conducted. Furthermore, the characteristic hemophagocytosis was not observed in his bone marrow tissue via bone marrow aspiration but some polymorphonuclear neutrophils were detected; Lack of lymphocytes was noted but there was no morphological evidence of malignancy (Fig. 1e, f). As a result, HPS was diagnosed according to the HLH-2004 diagnostic criteria. Then, the body temperature dropped to normal while the patient treated with intravenous Etoposide (40 mg, IV in one dose) and Dexamethasone (2 mg IV every 12 h).

Although his legal guardian, the parents, had been warned of the risks of the discharge, we failed to persuade them to continue to the treatment and the parents refused to accept further therapy due to the poor prognosis of HPS and X-SCID. Thus, the patient was discharged from our hospital after the parents signed an informed consent. At the time of discharge, splenomegaly and hepatomegaly were not improved, and papule on his trunk had not completely disappeared. He died 1 day after discharge.

**Fig. 2:**
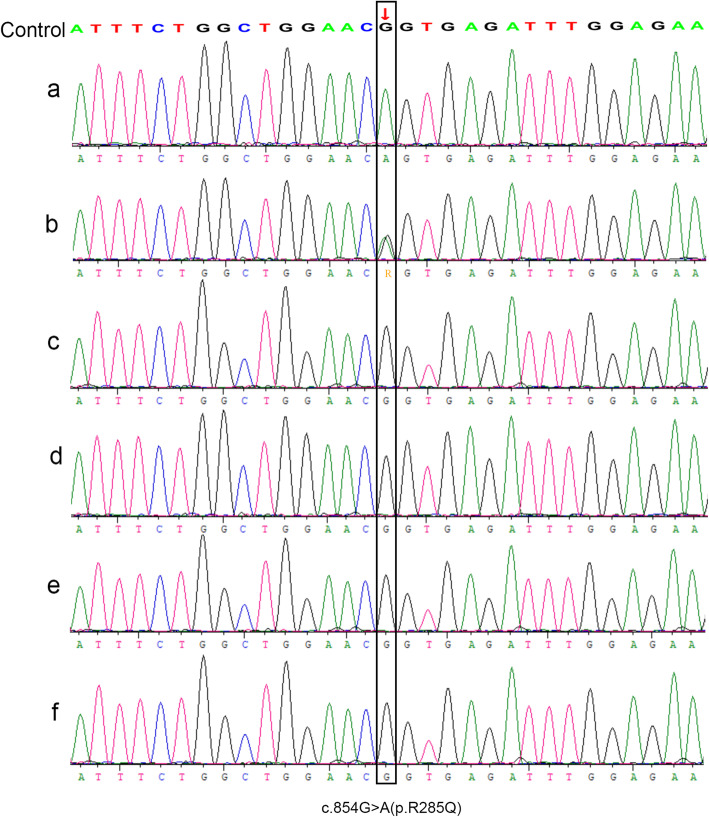
**a** Genetic detection of the mutation of in the *IL2RG* gene (Exon 6: c.854G > A; p.Arg285Gln) in the patient; **b** Genetic detection of the mother with respect to the mutation in the *IL2RG* gene (Exon 6: c.854G > A; p. Arg285Gln); **c** Genetic detection of the father was wild-type; **d** Genetic detection of the sister was wild-type; **e** Genetic detection of the maternal grandfather was wild-type; **f** Genetic detection of his maternal grandmother was wild-type

Another 18 days, the results of the genetic analysis revealed the mutation of *IL2RG* gene (Exon 6: c.854G > A; p.Arg285Gln) in the patient (Fig. 2a). Also, the genetic analysis of his mother showed the same mutation (Fig. 2b). However, the sister, father and maternal grandparents exhibited wild-type gene (Fig. 2c, d, e, f).

## Discussion and conclusion

Although the number of diagnosed secondary HPS cases was increased in recent years, the case of HPS due to *M. tuberculosis* complex, especially *M. bovis*, is yet rarely reported [2, 3]. Herein, we presented a boy admitted to PICU due to pneumonia, characterized by low lymphocyte counts, who was diagnosed with HPS associated with *M. bovis*. Finally, the patient was diagnosed as X-SCID by whole-exome sequencing. The finding suggested that peripheral pancytopenia and splenomegaly in the early stage of the disease, especially for the immunodeficiency patient, might be the cue to HPS. Moreover, in boys with low lymphocyte counts infected by *M. bovis*, X-SCID should be considered as diagnosis.

Hemophagocytosis was not observed in the bone marrow tissue via bone marrow aspiration (Fig. 1e). However, the symptoms, such as fever for ≥7 days over 38.5 °C, peripheral pancytopenia (Table 1), splenomegaly (Fig. 1d), increased ferritin in serum, elevated soluble CD25, and decreased fibrinogen fulfilled 6/8 HLH-2004 diagnostic criteria [1]. Thus, HPS was diagnosed in this patient. At present, HPS is almost uniformly fatal unless promptly recognized and treated with Etoposide and Dexamethasone [5]. Consequently, in the patient, the fever improved due to the treatment of Etoposide and Dexamethasone. Nonetheless, his parents refused to accept further therapy, and hence, further effect of the treatment could not be evaluated.

HPS is classified as primary and secondary. The results of genetic detection of the patient and his family members did not reveal any mutation in the *PFR1, UNC13D,* and *STX11* genes, which could lead to primary HPS [1]. Therefore, the possibility of primary-inherited HPS was excluded. On the other hand, although HPS due to *non-tuberculous Mycobacteria* as well as *M. tuberculosis* in adult have been reported, the report on HPS associated with *M. bovis* in children has not been reported [2, 6]. In this case, *M. bovis* was detected by mNGS and PCR-RDB, which was consistent with the strain of BCG vaccination in China [7]. Thus, the infection in the boy might be caused by BCG vaccination.

Previous studies showed that tuberculosis infection caused by BCG vaccination usually occurs in immunodeficient children [7, 8], especially for boys with low lymphocyte counts [9]. Due to the low lymphocyte counts, genetic analysis was performed, and the result of whole-exome sequencing confirmed the mutation in the *IL2RG* gene underlying X-SCID in the patient. Moreover, the genetic testing in his family members revealed that the mother was the carrier of the *IL2RG* mutation, but the patient’s maternal grandmother was the wild-type. Thus, we concluded that the mutation in the *IL2RG* gene might be inherited from the mother. It encodes common gamma chain (γc), which is a shared subunit of various cytokine (*IL-2, IL-4, IL-7, IL-9, IL-15,* and *IL-21*) receptors that are essential in lymphocyte development [10]. This might explain the low lymphocyte count in our patient. Several reports confirmed that the mutations in *IL2RG* gene lead to X-SCID, an immunodeficiency disease, characterized by low lymphocyte counts [4, 10, 11]. Some studies reported that immunodeficiency might be one of the risk factors related to HPS [12, 13].

In conclusion, a X-SCID in patient with *M. bovis*-associated HPS could be attributed to BCG vaccination. The symptoms, such as peripheral pancytopenia and splenomegaly might be the cue of HPS. Thus, X-SCID should be considered when *M. bovis* infection is detected in a male infant with low lymphocyte counts.

## Data Availability

Not applicable. All data generated or analyzed during this study are included in this published article.

## Abbreviations

*M. bovis*: *Mycobacterium bovis*
BCG: Bacillus Calmette-Guérin
HPS: Hemophagocytic syndrome
mNGS: Metagenomic next-generation sequencing
PCR-RDB: Polymerase Chain Reaction-Reverse Dot Blot
*IL2RG*: Interleukin-2 receptor subunit gamma
X-SCID: X-linked severe combined immunodeficiency
PICU: Pediatric Intensive Care Unit
bpm: Beats per minute
WBC: White blood cells
L: Lymphocyte
N: Neutrophil
PLT: Platelet
RBC: Red blood cells
HGB: Hemoglobin
CT: Chest computed tomography
CPAP: Continueous positive airway pressure
IVIG: Intravenous gammaglobulins
*M. tuberculosis complex*: *Mycobacterium tuberculosis complex*
ELISA: Enzyme-linked immunosorbent assay
H&E: Hematoxylin and eosin

## References

[1] Henter JI, Horne A, Aricó M, Egeler RM, Filipovich AH, Imashuku S, Ladisch S, McClain K, Webb D, Winiarski J (2007). HLH-2004: diagnostic and therapeutic guidelines for hemophagocytic lymphohistiocytosis. Pediatr Blood Cancer.

[2] Shi W, Jiao Y (2019). Nontuberculous Mycobacterium infection complicated with Haemophagocytic syndrome: a case report and literature review. BMC Infect Dis.

[3] Balkis MM, Bazzi L, Taher A, Salem Z, Uthman I, Kanj N, Boulos FI, Kanj SS (2009). Severe hemophagocytic syndrome developing after treatment initiation for disseminated Mycobacterium tuberculosis: case report and literature review. Scand J Infect Dis.

[4] Purswani P, Meehan CA, Kuehn HS, Chang Y, Dasso JF, Meyer AK, Ujhazi B, Csomos K, Lindsay D, Alberdi T (2019). Two unique cases of X-linked SCID: a diagnostic challenge in the era of newborn screening. Front Pediatr.

[5] Yanagisawa R, Nakazawa Y, Matsuda K, Yasumi T, Kanegane H, Ohga S, Morimoto A, Hashii Y, Imaizumi M, Okamoto Y (2019). Outcomes in children with hemophagocytic lymphohistiocytosis treated using HLH-2004 protocol in Japan. Int J Hematol.

[6] Misra S, Gupta A, Symes A, Duncan J (2014). Haemophagocytic syndrome after intravesical bacille Calmette–Guérin instillation. Scand J Urol.

[7] Li M, Chen Z, Zhu Y, Chen J (2019). Disseminated Bacille Calmette-Guérin infection in a patient with severe combined immunodeficiency caused by JAK3 gene mutation[J]. Pediatr Dermatol Pediatr Dermatol.

[8] Al-Hammadi S, Alsuwaidi AR, Alshamsi ET, Ghatasheh GA, Souid AK (2017). Disseminated Bacillus Calmette-Guérin (BCG) infections in infants with immunodeficiency. BMC Res Notes.

[9] van Vollenhoven R, Lee EB, Strengholt S, Mojcik C, Valdez H, Krishnaswami S, Biswas P, Lazariciu I, Hazra A, Clark JD (2019). Evaluation of the short-, mid-, and long-term effects of Tofacitinib on lymphocytes in patients with rheumatoid arthritis. Arthritis Rheumatol.

[10] Gray PE, Logan GJ, Alexander IE, Poulton S, Roscioli T, Ziegler J (2015). A novel intronic splice site deletion of the IL-2 receptor common gamma chain results in expression of a dysfunctional protein and T-cell-positive X-linked severe combined immunodeficiency. Int J Immunogenet.

[11] Clarke EL, Connell AJ, Six E, Kadry NA, Abbas AA, Hwang Y, Everett JK, Hofstaedter CE, Marsh R, Armant M (2018). T cell dynamics and response of the microbiota after gene therapy to treat X-linked severe combined immunodeficiency. Genome Med.

[12] Patiroglu T, Haluk Akar H, van den Burg M, Unal E, Akyildiz BN, Tekerek NU, Yilmaz E (2014). X-linked severe combined immunodeficiency due to a novel mutation complicated with hemophagocytic lymphohistiocytosis and presented with invagination: a case report. Eur J Microbiol Immunol (Bp).

[13] Tulloch LG, Younes R, Jeng A (2018). Reactive hemophagocytic syndrome in the setting of acute human immunodeficiency virus 1 infection: case report and review of the literature. Int J STD AIDS.

